# Magnetic Ionogel and Its Applications

**DOI:** 10.3390/gels11040219

**Published:** 2025-03-21

**Authors:** Sayan Ganguly, Shlomo Margel

**Affiliations:** 1 Department of Chemistry, University of Waterloo, 200 University Ave West, Waterloo, ON N2L 3G1, Canada; sayanganguly2206@gmail.com; 2 Department of Chemistry, Bar-Ilan Institute for Nanotechnology and Advanced Materials (BINA), Bar-Ilan University, Ramat-Gan 5290002, Israel

**Keywords:** magnetic ionogels, ionic liquids, smart materials, drug delivery systems, responsive actuators

## Abstract

Magnetic ionogels, a category of hybrid materials consisting of magnetic nanoparticles and ionic liquids, have garnered significant interest owing to their remarkable attributes, including tunability, flexibility, and reactivity to external magnetic fields. These materials provide a distinctive amalgamation of the benefits of both magnetic nanoparticles and ionogels, resulting in improved efficacy across many applications. Magnetic ionogels may be readily controlled using magnetic fields, rendering them suitable for drug administration, biosensing, soft robotics, and actuators. The capacity to incorporate these materials into dynamic systems presents novel opportunities for the development of responsive, intelligent materials capable of real-time environmental adaptation. Nonetheless, despite the promising potential of magnetic ionogels, problems persist, including the optimization of the magnetic particle dispersion, the enhancement of the ionogel mechanical strength, and the improvement of the long-term stability. This review presents a comprehensive examination of the syntheses, characteristics, and uses of magnetic ionogels, emphasizing significant breakthroughs and persistent problems within the domain. We examine recent advancements and prospective research trajectories aimed at enhancing the design and efficacy of magnetic ionogels for practical applications across diverse fields, including biomedical uses, sensors, and next-generation actuators. This review seeks to elucidate the present status of magnetic ionogels and their prospective influence on materials science and engineering.

## 1. Introduction

Magnetic gels, sometimes referred to as ferrogels, are a novel category of polymeric gel composites consisting of micro- or nano-sized magnetic particles interspersed within expanded polymeric gel matrices [1]. The combination of the diverse physical and mechanical properties of polymer and magnetic materials generates significant interest in these systems and their applications in numerous industrial and biomedical fields [2,3,4]. Magnetic gels are utilized for targeted medication delivery, industrial and biological sensors, and the fabrication of soft actuators and artificial muscles, as well as for cancer therapy, regenerative medicine, and tissue engineering [5,6]. The material’s viscoelastic qualities can be controlled by adjusting the kind and concentration of the polymer [7]. The attainment of a uniform dispersion of magnetic nanoparticles within a polymer continues to be a significant challenge [8,9,10]. Efforts to distribute a certain quantity of nanoparticles within a bulk polymer have demonstrated that the nanoparticles tend to agglomerate [11]. In a polymer solution, attempting the dispersion of nanoparticles significantly increases the likelihood of sedimentation, resulting in the non-uniform distribution of the material [12]. This work presents a method for achieving a uniform dispersion of nanoparticles in a polymer by the development of a gel, specifically by producing physical and chemical hydrogels/ferrogels [13,14]. The properties of ferrogel materials render them appropriate for various applications, including controlled medication release, mechanical devices, and artificial muscles [15,16,17]. The examination of the magnetic and elasto-magnetic characteristics, morphology, and nano-structure of ferrogels is presently the primary focus of researchers’ interest [13,18,19,20,21]. Iron oxide nanoparticles have numerous applications in medicine, including MRI diagnostics, targeted drug administration and controlled release, hyperthermia treatment of cancers, and magnetic biosensing [22].

Ionogels are a distinctive category of materials that merge the fluid characteristics of ionic liquids with the structural integrity of a gel matrix [23,24,25]. Ionogels are created by integrating ionic liquids—salts that remain liquid at ambient temperature—into a crosslinked polymer matrix, preserving the ionic conductivity and electrochemical stability of the ionic liquid while providing improved mechanical strength and flexibility [26]. These materials exhibit significant tunability, enabling the modification of attributes such as the mechanical flexibility, ionic conductivity, and electrochemical performance, rendering them suitable for use in energy storage systems, sensors, actuators, and flexible electronics [27]. Their elevated ionic conductivity and capacity for reversible shape alterations augment their attractiveness for applications in electronics necessitating responsive, adaptable functionality. Magnetic ionogels (MIGs) enhance the benefits of conventional ionogels by integrating magnetic nanoparticles, including iron oxide or ferrite-based particles, into the gel matrix. This enhancement confers magnetic responsiveness to the material, allowing it to respond to external magnetic fields [5]. MIGs integrate the superior ionic conductivity and flexibility of traditional ionogels with the capacity for dynamic manipulation in a magnetic field, rendering them suitable for applications in soft robotics, targeted drug delivery, and real-time bio-sensing [6,28,29]. The magnetic qualities enable the remote manipulation of mechanical attributes, including shape deformation and stiffness, hence promoting enhanced precision and adaptability in systems [30,31,32,33].

This review paper is innovative due to its thorough examination of MIGs, highlighting their distinctive integration of magnetic responsiveness and ionic liquid characteristics, which has not been comprehensively addressed in prior reviews. This paper synthesizes the distinct elements of magnetic nanoparticles and ionic liquids, offering a comprehensive understanding of their synergistic effects when integrated inside a gel matrix. This paper also emphasizes novel and insufficiently examined applications of MIGs, including sophisticated soft robotics, real-time bio-sensing and intelligent drug delivery systems, concentrating on their multifunctionality, adaptability, and responsiveness. This study provides a critical analysis of the current accomplishments, problems, and future directions, addressing a gap in the literature and presenting a novel perspective on the customization of MIGs for advanced technological applications.

## 2. Categorization of Ionogels

Ionogels can be roughly categorized into three types based on the supporting matrix and preparation techniques. Organic-polymer-based ionogels employ polymeric matrices, like poly(vinyl alcohol), poly(methyl methacrylate), or gelatin, to encapsulate the ionic liquid. Their adaptable structure and adjustable mechanical properties render them appropriate for use in flexible electronics and wearable sensors [34]. Inorganic-based ionogels are produced utilizing materials such as silica, alumina, or carbonaceous frameworks. These ionogels demonstrate exceptional thermal stability and are extensively utilized in catalysis, energy storage systems, and high-temperature applications [35]. Hybrid ionogels, which participate in both organic and inorganic matrices, provide superior mechanical strength, better conductivity, and greater functional diversity [36]. They are very efficacious in multifunctional applications, including bioelectronics and intelligent coatings [37]. Ionogels can be categorized according to their preparation techniques, such as sol–gel synthesis, physical gelation, and in situ polymerization, each affecting the structural characteristics and performance [38]. The ongoing advancement of new ionic liquids and creative matrix designs has broadened the applicability of ionogels in areas including medication delivery, environmental remediation, and sophisticated energy systems. This changing environment underscores the increasing importance of ionogels in both basic research and applied technology [39,40,41].

Ionogels are produced using several methods that facilitate the integration of ILs into a solid or semi-solid matrix while maintaining their distinguishing physicochemical characteristics. A prevalent technique is the sol–gel process, wherein a metal alkoxide precursor (e.g., tetraethyl orthosilicate, TEOS) experiences hydrolysis and condensation in the presence of an ionic liquid, resulting in a porous, gel-like network that encapsulates the ionic liquid inside its framework. This method is very beneficial for producing silica-based ionogels, which have elevated heat stability and adjustable mechanical characteristics. A prevalent technique is physical gelation, wherein polymers such as poly(vinyl alcohol) (PVA), poly(methyl methacrylate) (PMMA), or block copolymers encapsulate ionic liquids via non-covalent interactions, including hydrogen bonding or van der Waals forces, yielding flexible, self-healing ionogels appropriate for soft electronics and sensing applications. In situ polymerization is a significant method wherein monomers like acrylates or vinyl-based compounds are combined with an ionic liquid and polymerized via radical, UV, or thermal initiation. This technique facilitates exact regulation of the mechanical characteristics, rendering ionogels suitable for electrochemical devices, membranes, and energy storage applications. Moreover, electrospinning has emerged as an advantageous method for producing fibrous ionogels, wherein polymer solutions infused with ionic liquids are spun into nanofibers, resulting in materials with a high surface area and exceptional ionic conductivity. Supramolecular assembly techniques, like the integration of ionic liquids into cyclodextrins, metal–organic frameworks (MOFs), or peptide-based networks, enhance the design potential of ionogels by facilitating the creation of highly adjustable, self-assembled structures. Moreover, recent developments have investigated 3D printing and additive manufacturing to produce ionogels with intricate structures for bioelectronics, wearable sensors, and energy devices. Each synthesis method offers unique benefits regarding the mechanical strength, ionic conductivity, and processability, enabling the customization of ionogels for certain applications, including soft robotics and electrolyte membranes in batteries and supercapacitors. Ionogels can be categorized into multiple classifications according to their composition and synthesis methods, each providing distinct benefits for diverse applications (Table 1). Organic-polymer-based ionogels comprise ILs integrated within a polymer matrix, such as PMMA or PVA, rendering them suitable for flexible electronics and sensors. Ionogels generated from inorganic sol–gel processes employ silica or metal–oxide frameworks to encapsulate ionic liquids, offering superior thermal stability and appropriateness for electrolytes and supercapacitors. Hybrid ionogels, integrating organic and inorganic constituents, provide superior mechanical and electrochemical characteristics, rendering them advantageous for bioelectronics and energy applications. Self-assembled supramolecular ionogels utilize amphiphilic compounds, peptides, or block copolymers to create structured networks with ionic liquids, facilitating applications in drug delivery and biocompatible coatings. Electrospun fiber-based ionogels integrate ionic liquids into nanofiber matrices, enhancing ionic conductivity and mechanical integrity, rendering them advantageous for wearable sensors and membranes. Ultimately, 3D-printed and structurally designed ionogels utilize additive manufacturing methods to fabricate tailored structures for applications in soft robotics, smart fabrics, and biomedical devices. This classification highlights the adaptability of ionogels, enabling the customization of their features for particular technological innovations.

**Figure 1:**
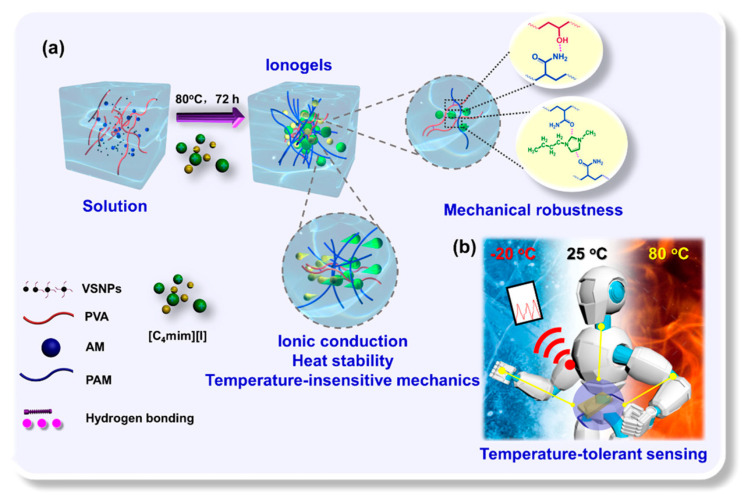
(**a**) Fabrication of tough, temperature-resistant, and conductive nanocomposite ionogels and (**b**) conductive nanocomposite ionogels may be utilized as sensors for monitoring both minor and significant human movements and temperature-resilient (−20 to 80 °C) pressure detection. Reprinted with permission from [63].

A high-performance wearable sensor with temperature-resistant mechanics and robust sensing capabilities was developed using hydrogen bonding between a poly(vinyl alcohol) (PVA)-infused nanocomposite interpenetrating network and the ionic liquid 1-butyl-3-methylimidazolium iodide ([C4mim][I]). The modification of hydrogen bonding and the resulting compatibility between [C4mim][I] and the network resulted in ionogels demonstrating enhanced mechanical properties, exceptional antifatigue performance, and sustained sensing capabilities throughout a broad temperature range [63]. The ionogel-based wearable sensor demonstrated consistent and reproducible sensitivity to diverse human movements, such as finger flexion, elbow joint flexion, and swallowing (Figure 1). Significantly, the pressure sensing capability may be entirely maintained within a service temperature range of −20 to 80 °C. This study presented a viable approach to developing a mechanically robust, thermally stable, ionogel-based multimode sensor, suitable for diverse applications such as electronic skins, human motion detection, and intelligent gadgets.

**Figure 2:**
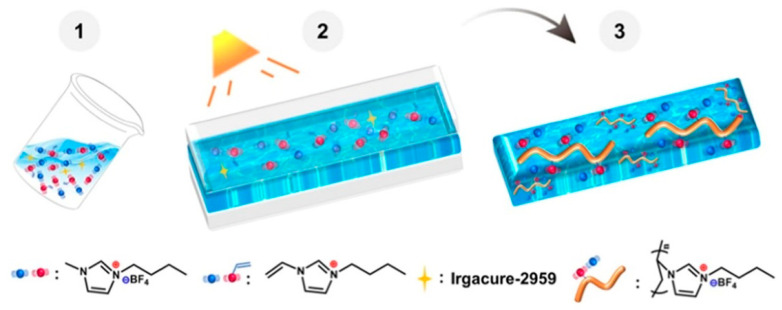
PIL/IL IG prepared by photoinitiation of [VBIm][BF_4_] in [BMIm][BF_4_]. Reprinted with permission from [65].

Physically crosslinked ionogels were developed through the random copolymerization of two monomers, acrylamide (AM) and N,N-dimethylacrylamide (DMAAm), in an ionic liquid. Utilizing the unique hydrogen bonding properties of the two monomers, we regulated the hydrogen bond content in the ionogel and examined its effects on the ionogel’s structure, transparency, and mechanical characteristics [64]. The resultant ionogel (Figure 2) exhibited exceptional mechanical properties, featuring a tensile strength of 8.94 MPa, an elongation at break of 404.4%, and a toughness of 21.94 MJ m^−3^ [65]. The ionogel demonstrated outstanding transparency and thermal stability, along with notable self-healing and shape memory properties. Furthermore, it could be readily recycled through sustainable means.

## 3. Magnetically Modified Ionogels (MIGs)

MIGs constitute a distinct category of functional materials that integrate the ionic conductivity of ionogels with the adjustable magnetic response of the included magnetic nanoparticles [66,67,68]. These materials are produced by integrating magnetic fillers, such iron oxide (Fe_3_O_4_), cobalt ferrite (CoFe_2_O_4_), or other superparamagnetic nanoparticles, into an ionogel matrix. The resultant composite demonstrates improved mechanical, electrical, and rheological characteristics, as well as sensitivity to external magnetic fields, rendering it an attractive option for applications in soft robotics, sensors, energy storage, and biomedicine. The mechanical properties of MIGs are significantly affected by the interactions between the polymeric or inorganic host matrix and the included magnetic fillers. The incorporation of magnetic nanoparticles often improves the modulus, toughness, and elasticity of the ionogel via nanoparticle–polymer interactions, hydrogen bonding, and physical crosslinking. The extent of the reinforcement is contingent upon the size, concentration, and uniformity of dispersion of the nanoparticles. External magnetic fields can dynamically modify the mechanical stiffness of the ionogel, facilitating applications in adaptive and reconfigurable materials [69]. Magnetically responsive MIGs have been utilized in shape-morphing soft actuators, where field-induced stiffening improves the load-bearing capacity. The electrical conductivity in MIGs is determined by the inherent conductivity of the ionic liquid phase and the percolation network established by the magnetic nanoparticles [70]. Research indicates that the integration of magnetic nanoparticles enhances the ion transport mechanism by altering the gel’s microstructure, resulting in improved ionic mobility. Excessive nanoparticle loading may diminish ionic conductivity by disrupting the ion channels in the gel matrix [71]. Utilizing conductive magnetic fillers, such as doped ferrites or carbon-coated iron oxides, can establish supplementary electrical conductivity routes, rendering MIGs suitable for flexible electronics and energy storage applications [72]. The rheological features of magnetorheological fluids are noteworthy due to their adjustable viscosity and shear-thinning characteristics when subjected to an external magnetic field. The alignment and clustering of magnetic nanoparticles under field influence modify the viscoelastic properties of the gel, resulting in regulated flow behavior [73]. This adjustable rheology is essential for 3D-printing applications, which necessitate precise regulation of the flow characteristics [32,74]. Furthermore, field-responsive viscosity modulation facilitates their application in magnetorheological dampers and adaptive tactile materials for haptic interfaces. The thermal characteristics of MIGs are considerably affected by the presence of magnetic nanoparticles. Numerous nanomaterials demonstrate elevated thermal conductivity, hence improving the heat dissipation in ionogel-based electronic devices [75]. Moreover, under alternating magnetic fields, MIGs can demonstrate localized heating effects attributed to nanoparticle-induced magnetothermal phenomena, which are advantageous for hyperthermia-based biomedical applications [36]. MIGs combine thermal stability with magnetic filler-induced heat generation, making them promising for thermal management.

**Figure 3:**
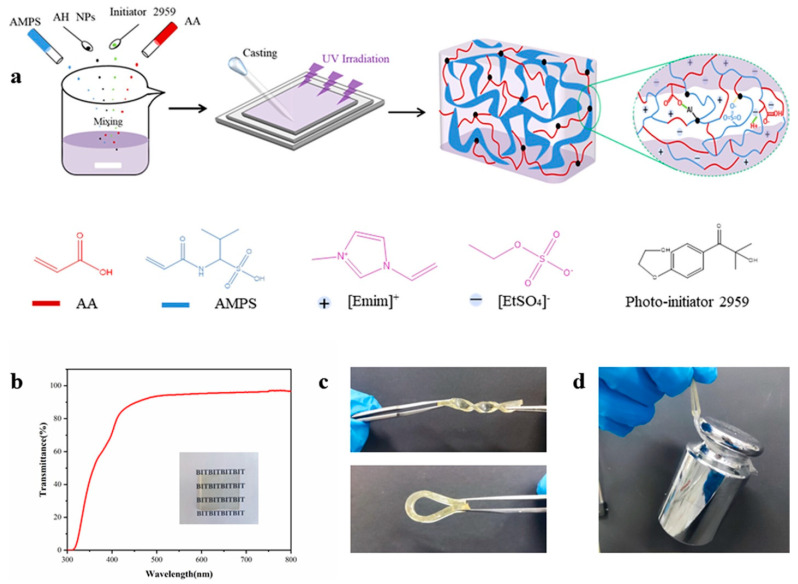
Preparation of the nanocomposite ionogels. (**a**) Schematic illustration of ionogel preparation via one-step photopolymerization. (**b**) Transmittance spectra of AP80Al12E with a film thickness of 1.7 mm. Illustration: photograph showing the high transparency of the film. (**c**) Photographs of an ionogel stretching and twisting. (**d**) Photograph of the ionogel (40 × 3 × 1.7 mm) supporting a 1 kg weight. Reprinted with permission from [75].

MIGs are synthesized using organosilane-coated iron oxide nanoparticles, N-isopropylacrylamide, and the ionic liquid trihexyl(tetradecyl)phosphonium dicyanamide. The ionogels synthesized using silane-modified nanoparticles exhibit greater homogeneity compared to those formulated with unmodified magnetite particles [76]. The silane-modified particles are immobilized within the ionogel and exhibit resistance to nanoparticle leaching. The altered particles also make the ionogels mechanically more stable than those generated with unaltered nanoparticles. The ionogels react to external permanent magnets, therefore serving as prototypes for a novel soft magnetic actuator. Shojaee et al. documented the utilization of environmentally benign solvents, including water, deep eutectic solvents, and ionic liquids, in diverse organic transformations [77]. We provide a novel and straightforward sol–gel approach to simultaneously encapsulate ionic liquid and magnetic nanoparticles within a polymer matrix via a single-step procedure [78]. The fundamental concept established is clear, pragmatic, and relevant to the industrial revolution owing to its eco-friendly characteristics and cost-effective raw materials. Yuan et al. delineated a magnetic nanoparticle drug carrier for regulated drug release that reacts to variations in the external temperature or pH, exhibiting a prolonged circulation duration and diminished adverse effects. The innovative nanocarrier features a functionalized magnetite (Fe_3_O_4_) core coupled to a drug by an acid-labile hydrazone bond and encased by the thermosensitive smart polymer chitosan-g-poly(N-isopropylacrylamide-co-N,N-dimethylacrylamide) [chitosan-g-poly(NIPAAm-co-DMAAm)] [79]. The chitosan-g-poly(NIPAAm-co-DMAAm) smart polymer demonstrates a reduced critical solution temperature (LCST) of approximately 38 °C, indicating phase transition behavior and facilitating its application in on–off mechanisms. The drug release response is much lower at temperatures below the LCST compared to those above the LCST. The iron-based ionic liquid (IL) 1-butyl-3-methylimidazolium tetrachloroferrate(III) [Bmim][FeCl_4_] has served as a precursor in the fabrication of transparent, ion-conductive, and paramagnetic ionogels. UV/Vis spectroscopy indicates that the coordination environment of the Fe(III) ion undergoes modest alteration during the integration of the ionic liquid into PMMA. The thermal stability of PMMA markedly improves with the introduction of ionic liquids. The first weight loss noted at approximately 265 °C for pure PMMA is entirely inhibited [80]. The ionic conductivity exhibits significant temperature dependency and rises with higher ionic liquid weight percentages. The magnetic characteristics resemble those documented for the pure ionic liquid and remain unaffected by integration into the PMMA matrix. The resultant ionogel serves as a compelling prototype for soft, flexible, and transparent materials that integrate the mechanical properties of the matrix with the functionalities of the metal-containing ionic liquid, including magnetism. The dual electro- and magneto-responsive material is synthesized from a physically crosslinked agarose ionogel infused with magnetite nanoparticles (Fe_3_O_4_ NPs). The primary investigation is the bending reactions induced by the dielectrophoretic force from the AG electric dipoles, as well as the ion migration under an applied electric field, and the magnetic attraction and movement of magnetic particles under an applied magnetic field [81]. The Fe_3_O_4_ nanoparticles are initially generated using a surfactant-assisted co-precipitation process, followed by their incorporation into an agarose solution to create the magnetic ionogel (Fe_3_O_4_/AG MagIGel) by a solution-casting technique. The impact of the Fe_3_O_4_ nanoparticle concentrations on the Fe_3_O_4_/AG MagIGels was examined regarding the structural, thermal, magnetic, dielectric, electrical conductivity, and rheological properties. An ionogel was synthesized through the selection of suitable polymer monomers, ionic liquids, crosslinkers, and photoinitiators (Figure 3) [75]. The produced precursor solution had a pale-yellow hue and demonstrated excellent flowability and transparency. The compatibility of the monomers with EMIES produced exceptional uniformity in the synthesized ionogels, guaranteeing their optical transparency. Figure 3b illustrates that the AP80Al12E gel, measuring 1.7 mm in thickness, had exceptional transparency, with an average transmittance of 93% in the visible light spectrum. The establishment of these hydrogen bonds confines the ionic liquid within the gel network, reducing leakage and enhancing the structural integrity of the ionogel. The produced ionogel samples have exceptional flexibility and mechanical qualities, evidenced by their capacity to twist, bend (Figure 3c), and sustain a 1 kg weight (Figure 3d).

**Figure 4:**
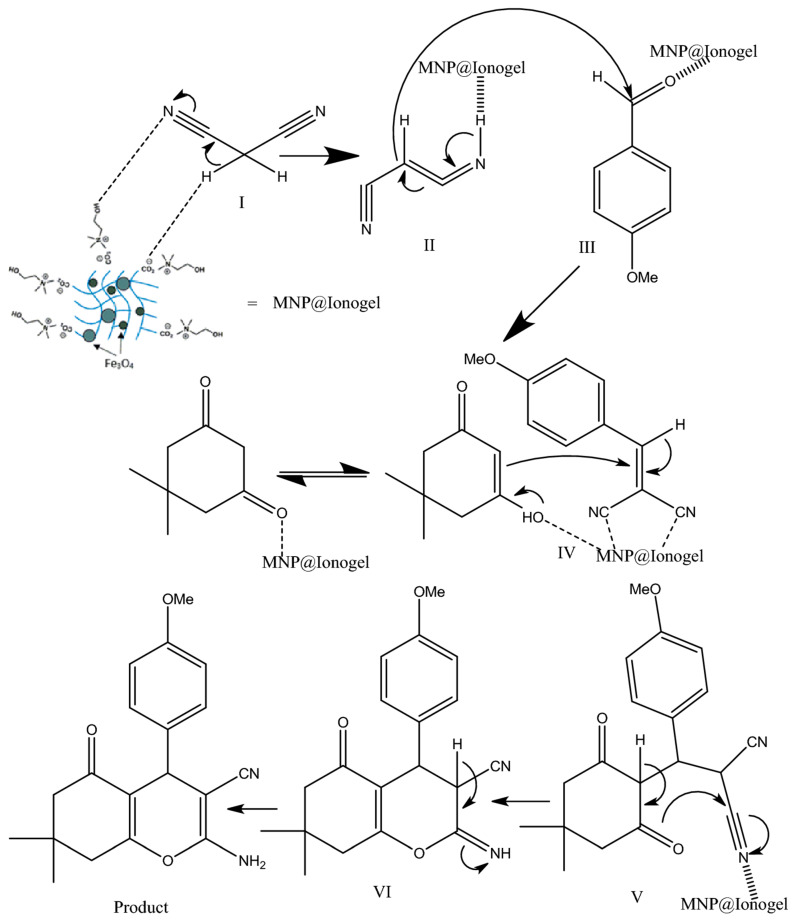
The proposed reaction mechanism. Reprinted with permission from [77].

Based on the proposed mechanism, we posit that the synthesized magnetic ionogel catalyst facilitates both the Knoevenagel condensation reaction and the Michael addition [77]. The nucleophilic addition of an activated compound to a carbonyl group (intermediates II–III), followed by a Michael addition (intermediate IV) of a nucleophile to an α,β-unsaturated carbonyl compound, has resulted in the formation of the product (Figure 4).

## 4. Applications of MIGs

Magnetic ionogels, a distinctive category of soft materials that integrate ILs and magnetic nanoparticles (MNPs) within a gel matrix, have emerged as a potential material for diverse applications owing to their adjustable mechanical, electrical, and magnetic characteristics. These hybrid materials demonstrate the fluidity and elevated ionic conductivity of ionic liquids while preserving the structural integrity of a gel, rendering them appropriate for applications in soft robotics, energy storage, biomedicine, and environmental remediation. Magnetic ionogels are being increasingly investigated in soft robotics for their capacity to react to external magnetic fields without requiring direct physical interaction. These materials can be designed to demonstrate programmed deformation, facilitating shape transformation, self-healing, and regulated movement. Magnetic ionogel-based actuators and artificial muscles are especially appealing for biomedical applications, where soft, flexible materials are required for minimally invasive devices. Moreover, their self-repairing properties guarantee longevity, rendering them suitable for extended robotic applications. The elevated ionic conductivity and electrochemical stability of magnetic ionogels render them excellent candidates for energy storage devices, such as supercapacitors and lithium-ion batteries. The use of magnetic nanoparticles in these ionogels can augment the charge transfer and enhance the energy density. Moreover, the incorporation of ionic liquids inhibits volatilization and improves thermal stability, rendering them safer substitutes for traditional liquid electrolytes in batteries and capacitors. Magnetic ionogels are under investigation for uses in drug delivery, tissue engineering, and biosensing. Their biocompatibility and regulated magneto-responsive properties enable their employment in targeted drug delivery, wherein medicines are contained within the ionogel and released upon the application of an external magnetic field. Furthermore, these ionogels can serve as scaffolds in tissue engineering, as their porous architecture and adjustable mechanical properties facilitate cell growth and differentiation. Magnetic ionogels are included in biosensors for biomolecule detection, where their ionic conductivity and magnetic responsiveness improve the sensitivity and detection thresholds. Magnetic ionogels are crucial for environmental remediation, especially in the extraction of heavy metal ions and organic contaminants from water. Their extensive surface area, chemical adaptability, and magnetic characteristics enable efficient pollutant adsorption and facilitate straightforward separation from the environment via external magnetic fields. These properties make them excellent candidates for wastewater treatment and oil spill cleanup, where reusable, magnetically retrievable absorbents are required. Magnetic ionogels are utilized in coatings to create intelligent surfaces that react to environmental stimuli, like the temperature, light, or magnetic fields. These coatings are employed in anti-corrosion applications, anti-fouling surfaces, and adaptive optical films. Furthermore, they can be integrated into wearable gadgets and sensors for real-time environmental monitoring, as their magnetic and conductive features facilitate multifunctionality.

**Figure 5:**
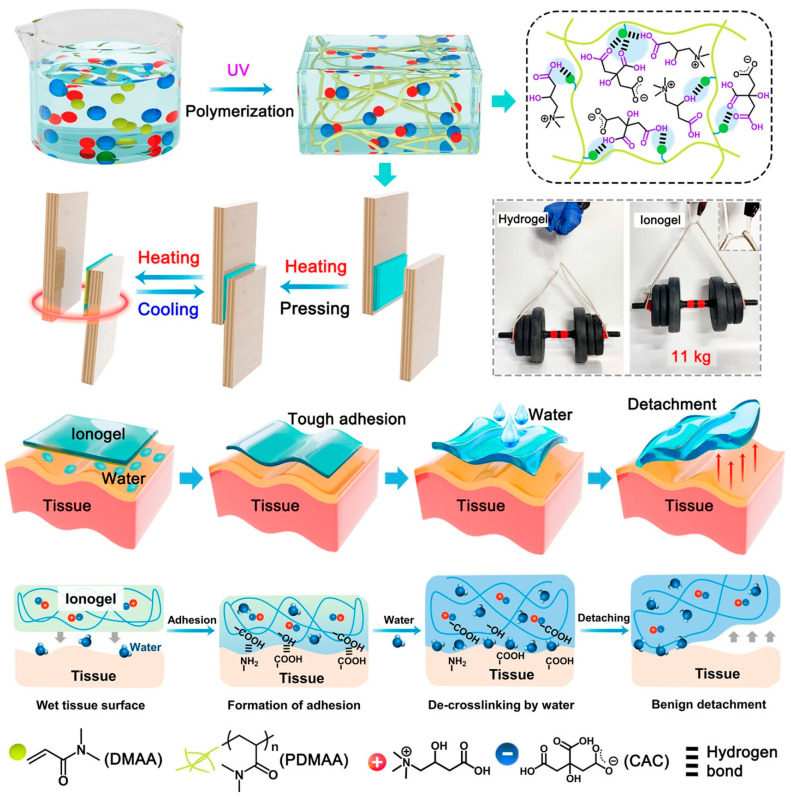
Ionogel structure and supramolecular interaction. Ionogels were made by polymerizing CAC and DMAA. The liquids and polymer chains formed a dense supramolecular network due to the number of hydrogen bonding sites, creating a robust ionogel (CAC content: 45 wt%) that can bear 11 kg. The generated ionogel balances intermolecular cohesion energy with interfacial adhesion energy, generating a strong adhesion with substrates and allowing heating/cooling. Hydrogen bonding interactions can crosslink the ionogel to the tissue surface for mechanically matched strong adherence. Finally, the damaged reversible bonds allow a saline solution to gently remove the ionogel [89].

Ionogel sensors have garnered significant interest as an alternative to hydrogel sensors, as they are promising materials for addressing the issues of rapid drying and freezing [82]. Nevertheless, the inadequate mechanical qualities of ionogels have significantly impeded their widespread deployment [83]. A resilient physically interconnected double-network ionogel (DN ionogel) was synthesized by interpenetrating a poly(hydroxyethyl acrylate) network within an agarose network in 1-ethyl-3-methylimidazolium chloride [84]. The application of this class of nanocomposites is currently hindered by many limitations, mostly due to the disparity between the mechanical characteristics of the polymer and the metallic electrodes, which undermines their stability and durability during cyclic deformation. Santaniello et al. documented the application of supersonic cluster beam implantation (SCBI) as a proficient method to produce soft electroactive ionic polymeric nanocomposite actuators. SCBI utilizes supersonically accelerated beams of neutral metal nanoparticles that can be aerodynamically collimated and directed onto a polymeric substrate to produce thin nanostructured metal layers that physically interpenetrate the polymer [85]. Ionogels have received significant interest as stretchy conducting materials. It is essential to incorporate various functions, including mechanical robustness, room temperature self-healing ability, ease of processing, and recyclability, into an ionogel-based device with significant potential for applications like soft robotics, electronic skins, and wearable electronics. This study proposes a multilayer hydrogen bonding technique, inspired by the structure of spider silk, to effectively manufacture multifunctional ionogels with a combination of desirable features. The ionogels are made using N-isopropylacrylamide (NIPAM), N,N-dimethylacrylamide (DMA), and the ionic liquid 1-ethyl-3-methylimidazolium bis(trifluoromethylsulfonyl)imide ([EMI][TFSI]). The synergistic hydrogen bonding interactions among the PNIPAM chains, PDMA chains, and ILs confer enhanced mechanical strength and rapid self-healing capabilities to the ionogels under ambient conditions [86]. Carbonyl-rich poly(ethylene-glycol-adipate) diols are chosen as soft segments to offer ample interaction sites for ionic liquids, hence imparting great transparency, stretchability, and elasticity to the ionogel. A self-healing ionogel with a tensile strength of 1.65 ± 0.08 MPa has been successfully generated, surpassing all the previously reported transparent room-temperature self-healing ionogels, and its implementation in a 3D-printed stretchable numeric keyboard was demonstrated [87]. Researchers investigated the dissolving of agarose utilizing low-viscosity alkyl or hydroxyalkyl ammonium formate ILs. The combination of these ionic liquids with imidazolium or pyridinium ionic liquids improved the dissolving of agarose. The dissolved agarose was either reconstituted with methanol or transformed into ionogels by cooling the agarose–IL solutions to room temperature. Ionogels produced from N-(2-hydroxyethyl)ammonium formate or their combination with 1-butyl-3-methylimidazolium chloride have shown remarkable strength. These robust, highly conductive ionogels have been showcased in applications like electrochromic windows and possess potential for more soft matter electrical devices and biomedical applications. Researchers synthesized a poly(acrylamide-co-acrylic acid) [P(AM-co-AA)] ionogel using chemical polymerization, attaining optical transparency and flexibility. The polymer network efficiently regulated the ion dispersion, yielding unique dielectric characteristics appropriate for electromagnetic metamaterials. Two devices were developed: a broadband absorber achieving over 90% absorption between 10.9 and 25.36 GHz, and a frequency-selective absorber that transmits from 4.62 to 5.86 GHz, exhibiting negligible insertion loss and over 90% absorption from 13 to 17.8 GHz. The experimental outcomes corresponded closely with the predictions, underscoring the promise of ionogels in flexible electromagnetic metamaterial applications [88]. Xiong et al. have created a conductive ionogel utilizing deep eutectic solvents (DESs) that demonstrates exceptional transparency, stretchability, little hysteresis, and tunable adhesion [89]. These characteristics render it appropriate for mechanosensing applications in both aerial and aquatic environments (Figure 5). The ionogel’s distinctive amalgamation of mechanical and conductive properties facilitates its application in flexible sensors, underscoring its promise across diverse domains, such as wearable technology and underwater monitoring systems.

**Figure 6:**
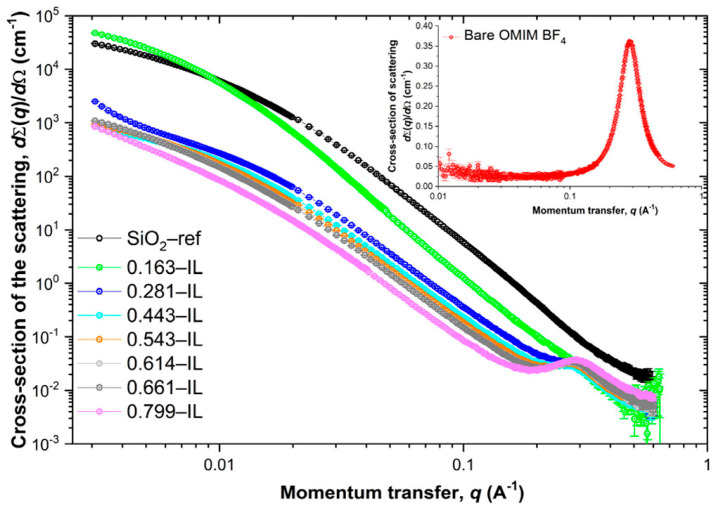
SAXS data for the OMIM BF_4_@SiO_2_ ionogels, SiO_2_-ref sample and bare OMIM BF4 ionic liquid (inset). Reprinted with permission from [93].

In another study, researchers created ionogels by encapsulating the ionic liquid Aliquot 336 within a silica matrix. Thorough characterization validated the effective encapsulation of the ionic liquid within the silica matrix. The work elucidated the mechanics of the iron(III) extraction, emphasizing the function of the confined ionic liquid in enhancing the extraction efficiency. These findings indicate possible uses of these ionogels in the extraction and separation of metal ions [90]. Researchers produced an innovative conductive textile by applying a liquid metal coating on cloth and enclosing it with a poly(acrylamide-co-acrylic acid) ionogel. This composite material demonstrated superior electromagnetic interference (EMI) shielding efficacy, with a peak of 49.3 dB for a single layer and an average of 73.0 dB for three layers. The material exhibited low reflectivity (0.404), signifying negligible secondary reflection of the electromagnetic waves [91]. The ionogel encapsulation increased the fabric’s tensile strength to 13.16 MPa and ensured stability across a broad temperature range (−18 to 100 °C). These characteristics indicate possible uses in wearable electronics and protective apparel, providing efficient EMI shielding while preserving flexibility and durability. In a separate work, the researchers synthesized magneto-iono-elastomers (MINEs) by incorporating a magnetic ionic liquid, [Emim][FeCl_4_], into a urethane-based polymer matrix. This design efficiently restricts the magnetic anions via robust intermolecular interactions, such as possible hydrogen bonds and metal-coordination bonds, facilitating a substantial ionic liquid loading of up to 80 wt% while preserving the structural integrity [92]. The resultant MINEs demonstrate remarkable magnetization (2.6 electromagnetic units per gram), elevated ionic conductivity (more than 10^−3^ S/cm), hyperelasticity with elastic recovery reaching 99%, and self-healing properties. The characteristics of MINEs render them suitable for several magnetoelectronic applications, including wearable strain sensors, contactless magneto-responsive electronics, transparent touch panels, and flexible magnetic carriers. Yu et al. created ionogels by infusing Aerosil A380 silica with different concentrations (16.3 to 79.9 mol%) of the ionic liquid 1-methyl-3-octyl-imidazolium tetrafluoroborate (OMIM BF_4_). Characterization techniques, such as ^19^F NMR spectroscopy, demonstrated shifts and broadenings in the BF_4_^−^ signal, signifying confinement effects within the silica matrix. A thermal study revealed a substantial reduction (about 50 °C) in the breakdown temperature of the ionic liquid when confined. Infrared spectroscopy corroborated these interactions by revealing modified vibrational frequencies. Textural research revealed that the ionic liquid sequentially filled the micropores, mesopores, and interparticle voids inside the silica. Small-angle X-ray scattering (SAXS) was utilized to examine the structural arrangement of the confined ionic liquid. The SAXS tests indicated an increase in the dimensions of the nonpolar domains from 21.5 Å in pure OMIM BF_4_ to 25.6 Å in ionogels with 28.1 mol% ionic liquid. In ionogels with minimal ionic liquid content (16.3 mol%), the absence of nonpolar correlations indicates considerable structural distortion resulting from confinement (Figure 6). The SAXS results indicated that the ionic liquid domains reformed based on the loading, offering essential insight into the influence of confinement on the nanostructure of ionogels. This study emphasizes that structural manipulation using SAXS analysis can enhance the ionogel characteristics for specific applications.

The authors investigated the potential of ionogels—gels that integrate ionic liquids—as novel solutions in biological applications. They emphasized the distinctive characteristics of ionogels, including the elevated ionic conductivity, adjustable mechanical strength, and intrinsic antibacterial capabilities, rendering them appropriate for wound-healing and drug delivery applications [94]. The study examined current progress in the development of ionogels that facilitate tissue regeneration, enable regulated drug release, and improve the bioavailability of medicinal drugs. The authors discussed contemporary issues, such as biocompatibility and the necessity for comprehensive clinical studies, while highlighting encouraging advancements, like the FDA approval of MRX-5LBT^®^, a transdermal drug delivery system, which will facilitate future clinical applications of ionogels.

## 5. Toxicity of Ionogels

**Figure 7:**
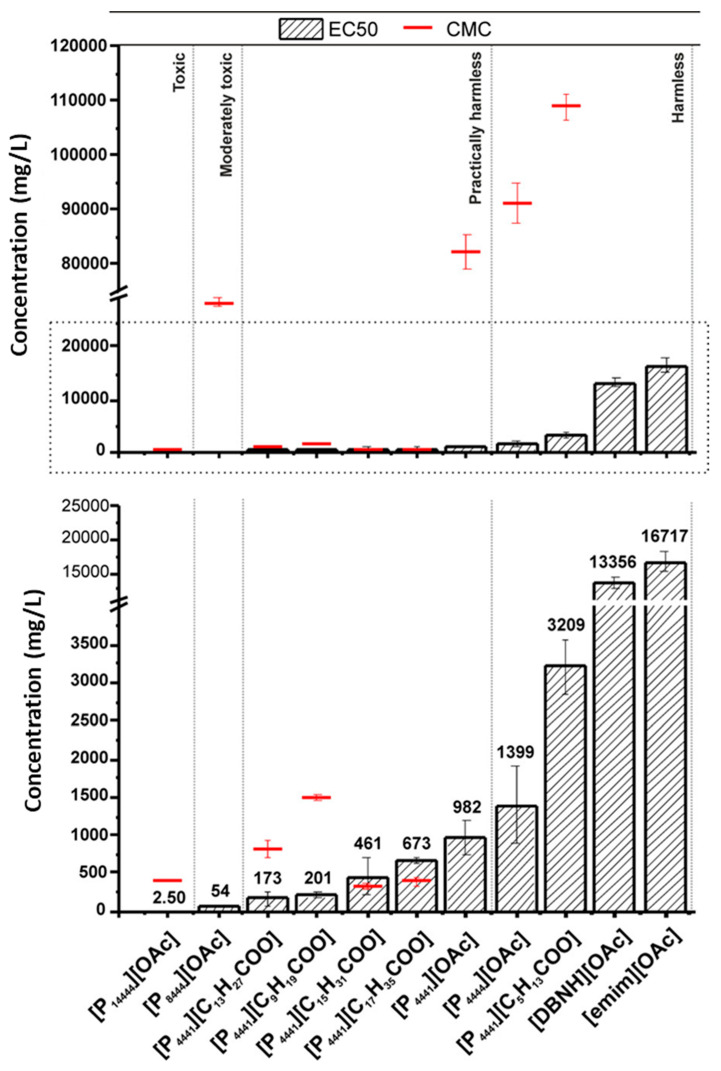
IL cellular toxicity on CHO cells—EC50s and CMCs. A 2 h alamarBlue assay was used to assess the cytotoxicity after 24 h of incubation with the identified ILs at varying doses. Upper figure’s designated area is increased in the lower figure [101].

The toxicity of ionogels is a crucial factor to evaluate, particularly for their uses in the biomedical and environmental domains. Ionogels, consisting of ILs and a solid matrix (often polymeric), are progressively utilized in many fields, including medicine administration, sensors, and energy storage [95]. The distinctive characteristics of ILs employed in ionogels—namely their low volatility, elevated ionic conductivity, and stability—prompt concerns over their biocompatibility and possible toxicity [35]. Comprehending the toxicological characteristics of ionogels is crucial to guaranteeing their safe use, especially in medical and consumer applications. Ionic liquids demonstrate various biological effects contingent upon their chemical composition, which might differ markedly based on the selected cations and anions [94]. Certain ionic liquids are regarded as highly poisonous, whilst others demonstrate reduced toxicity levels. Toxicity may be affected by parameters like the chemical structure of the ionic liquid, concentration, duration of exposure, and exposure route (e.g., dermal contact, inhalation, or ingestion). The toxicity of ionic liquids may be modified by their interaction with the matrix material when integrated into ionogels. The matrix may affect the release rate of the ionic liquid, the diffusion of hazardous constituents, or the breakdown products that may be detrimental to living beings [96]. Numerous research studies have examined the cytotoxicity of ionogels, especially concerning their application in drug delivery systems or bioelectronics. Cell viability assays, including MTT (3-(4,5-dimethylthiazol-2-yl)-2,5-diphenyltetrazolium bromide) and Live/Dead staining, are frequently employed to evaluate the effects of ionogels on cultured cells. These experiments often demonstrate that ionogels composed of specific ionic liquids exhibit dose-dependent cytotoxicity, with increased concentrations of ILs resulting in substantial decreases in cell viability. The toxicity level frequently relies on the specific ionic liquid utilized in the ionogel and the concentration of the ionic liquid within the gel matrix [97]. The direct toxicity of ionogels is significant, but the matrix material also influences the total toxicity, either alleviating or exacerbating it [98]. Polymeric matrices, including PVA, gelatin, and silica-based materials, exhibit differing degrees of biocompatibility. Biodegradable polymers may pose diminished danger, as they decompose gradually within the body, hence mitigating the risk of buildup and chronic toxicity. Conversely, non-biodegradable matrices may present a heightened risk if the ionogel degrades gradually, releasing the ionic liquid over time and resulting in extended exposure to potentially deleterious compounds. A principal worry regarding the toxicity of ionogels is their propensity to elicit immune system reactions. In biomedical applications, particularly in medication administration or as implants, ionogels may interact with the body’s immune system [99]. If the ionic liquid in the ionogel leaches into the bloodstream, it may elicit an inflammatory response or impair cellular function, resulting in tissue damage. The polymeric matrix may affect the immune system’s perception of the ionogel, either augmenting or diminishing immunological recognition. Alongside direct cellular toxicity, environmental toxicity is a significant concern when ionogels are discarded or undergo degradation over time. The introduction of ionic liquids into the environment may threaten aquatic organisms and ecosystems, particularly due to their high water solubility and persistence. This environmental issue highlights the necessity of creating ionogels with biodegradable matrices and eco-friendly ionic liquids, guaranteeing that their final disposal does not adversely affect ecosystems. Zhang et al. demonstrated that the most hazardous ionic liquid is [C12mim]NO_3_, while the least toxic ionic liquid is [C2mim]NO_3_, as assessed on *Danio rerio*. The LC50 values for ionic liquids with various anions were comparable. We contend that ionic liquids with varying alkyl chain lengths exert a more significant impact on acute toxicity to aquatic species than other anions [100]. The median effective concentrations (EC50s) and critical micelle concentrations (CMCs) were assessed to elucidate the relationship between IL aggregation and the toxicity of ILs [101]. The long-chain ionic liquids were markedly more harmful than their short-chain counterparts, with the length of the anion chain proving to be less significant than that of the cation chain in evaluating the effects of ionic liquids on organism viability (Figure 7). Moreover, the majority of the ILs existed as monomers at the point when the EC50 was attained. The ionic liquids employed in the long-term testing exhibited no notable impact on zebrafish behavior, reproduction, or histology within the tested concentration range.

The impact of ionic liquids on bacterial cells, as determined by specific interactions related to cell wall construction, was assessed [102]. The effect was evaluated by viability assays and scanning electron microscopy observations. The results demonstrated that ionic liquids display varying degrees of toxicity toward Gram-positive and Gram-negative microorganisms. The effects are linked to the chemical structure of the cationic species of the ionic liquids and their critical micelle concentration value. The presence of an alkyl or allyl group was found to enhance toxicity, while the inclusion of an aryl group in the cation diminished the poisonous effect of ionic liquids. This study’s results also demonstrated unforeseen impacts of *E. coli* cell self-aggregation. The investigated ILs showed increased toxicity against Gram-positive bacteria due to varying interactions with the cell membranes. Prior to the probable industrial discharge of ILs into the environment, it is essential to assess their toxicological and antimicrobial characteristics. To assess the microbiological toxicity of imidazolium and pyridinium ionic liquids with differing alkyl chain lengths, Docherty et al. studied *Vibrio fischeri* utilizing the Microtox assay [103]. An augmentation in the alkyl group chain length and the quantity of the alkyl groups substituted on the cation ring correlated with heightened toxicity.

## 6. Conclusions and Future Perspective

Magnetic ionogels, distinguished by their integration of magnetic particles and ionic liquids, have emerged as a potential category of materials owing to their exceptional features, such as their increased flexibility, tunability, and responsiveness to external magnetic fields. These materials possess considerable potential in several applications, such as medication delivery systems, sensors, actuators, and soft robotics, due to their capacity to respond dynamically to magnetic stimuli. Notwithstanding the advancements achieved, issues such as the optimization of magnetic particle dispersion, the mechanical characteristics of ionogels, and their long-term stability require resolution for wider use. Subsequent research needs to concentrate on refining the synthesis techniques, augmenting the material characteristics, and investigating innovative applications that leverage the distinctive capabilities of magnetic ionogels in intricate settings. Owing to the ongoing improvements, magnetic ionogels are set to become essential components of next-generation smart materials, providing novel solutions in biomedical engineering, electronics, and materials science.

The future of magnetic ionogels is highly promising, owing to their distinctive amalgamation of ionic liquid characteristics and magnetic capabilities. The potential of magnetic ionogels for biomedicine, energy storage, and environmental sensing is substantial; yet, their practical implementation depends on addressing critical issues concerning scalability, biocompatibility, and sustainability. A primary focus for future research is the advancement of sustainable and eco-friendly synthesis techniques for magnetic nanoparticles and ionogels. Current synthesis methodologies prioritize efficiency and performance, although there is an increasing focus on “green” techniques that mitigate environmental effects, including the use of non-toxic solvents and renewable raw materials, and the reduction of energy use. This would correspond to the overarching sustainability trends in materials science and might substantially augment the attractiveness of magnetic ionogels for biomedical and environmental applications. Although ionogels have potential for regulated drug release and tissue regeneration, their prolonged contact with biological systems raises concerns. Improvements in the surface modification and the use of biodegradable matrices may resolve these challenges, resulting in safer and more effective therapeutic applications. Furthermore, comprehensive investigations of their toxicity and immunological responses are essential to evaluate the viability of employing ionogels in clinical applications, especially for purposes such as implanted devices or prolonged drug administration. Magnetic ionogels may be utilized in soft robotics and actuation systems, where their magnetic responsiveness may be exploited for meticulous control of movement and force. The creation of multifunctional ionogels possessing both magnetic and non-magnetic characteristics will broaden their use, possibly introducing novel domains in adaptive materials, responsive coatings, and energy harvesting. Scalability and repeatability difficulties continue to pose substantial obstacles to the extensive implementation of magnetic ionogels in industrial settings. Future research should concentrate on refining the synthesis pathways to enhance the cost-effectiveness and scalability, ensuring the consistent production of high-quality materials. Moreover, standardized characterization techniques and creation of performance benchmarks would be essential for assessing and comparing the efficacy of diverse magnetic ionogels across various applications.

## Figures and Tables

**Table 1:** Types and applications of ionogels.

Classification	Description	Examples	Applications	Ref.
Organic-Polymer-Based Ionogels	Ionogels where ionic liquids (ILs) are entrapped within a polymer matrix. The polymer can be physically or chemically crosslinked.	PMMA-IL ionogels, PVA-IL ionogels	Flexible electronics, sensors, energy storage	[42,43,44,45]
Inorganic Sol–Gel-Derived Ionogels	ILs are encapsulated in an inorganic silica or metal–oxide network via sol–gel chemistry.	Silica-IL ionogels, alumina-IL ionogels	Electrolytes, catalysis, supercapacitors	[43,46,47]
Hybrid Ionogels	Combination of organic polymers and inorganic networks to enhance mechanical and electrochemical properties.	Silica-PMMA-IL ionogels, MOF-IL hybrids	Advanced energy materials, bioelectronics	[48,49,50,51]
Self-Assembled Supramolecular Ionogels	Ionogels formed by molecular self-assembly of amphiphilic molecules, peptides, or block copolymers with ILs.	Peptide-IL ionogels, cyclodextrin-IL gels	Drug delivery, biocompatible coatings	[52,53,54,55]
Electrospun Fiber-Based Ionogels	ILs incorporated into electrospun nanofiber networks to enhance conductivity and mechanical strength.	PEO-IL nanofiber ionogels	Wearable sensors, high-performance membranes	[56,57]
3D-Printed and Structurally Engineered Ionogels	Ionogels fabricated via additive manufacturing for customized architectures.	3D-printed ionogels using SLA or DIW	Soft robotics, smart textiles, biomedical devices	[58,59,60,61,62]

## Data Availability

No new data were created or analyzed in this study. Data sharing is not applicable to this article.
